# Integration of Transcriptomic and Proteomic Analyses Reveals New Insights into the Regulation of Immune Pathways in Midgut of *Samia ricini* upon SariNPV Infection

**DOI:** 10.3390/insects13030294

**Published:** 2022-03-16

**Authors:** Gang Li, Benzheng Zhang, Huan Zhang, Anying Xu, Heying Qian

**Affiliations:** 1 The Sericultural Research Institute, Jiangsu University of Science and Technology, Zhenjiang 212003, China; zbzheng3399@163.com (B.Z.); srixay@126.com (A.X.); 2 State Key Laboratory of Integrated Management of Pest Insects and Rodents, Beijing 100101, China; zhanghuan@ioz.ac.cn

**Keywords:** *Samia ricini*, SariNPV, transcriptomics, proteomics, infection mechanism

## Abstract

**Simple Summary:**

SariNPV is one of the main pathogens of *Samia ricini* and its infection of *Samia ricini* sericulture has caused significant economic losses to society. In this study, we aim to reveal the molecular mechanism of pathogen–host interactions in SariNPV-infected *S. ricini* through transcriptomic and proteomic analyses. Using RNA-sequencing and iTRAQ, we mapped the differentially expressed genes (DEGs) and proteins (DEPs) that are involved in the immune responses of *S. ricini* upon virus invasion. Based on our analyses, we identified specific DEGs and DEPs that are involved in various essential biological signaling pathways and immune-related pathways upon SariNPV invasion. These DEGs and DEPs play an important role in triggering host immune responses to pathogens. Our study provides molecular insights into host immune responses regarding pathogen invasion, in particular, the immune response mechanism and network of *S. ricini* in response to SariNPV infection.

**Abstract:**

*Samia ricini* nucleopolyhedrovirus (SariNPV) is one of the main pathogens of *S. ricini* sericulture and its infection causes severe impacts on economic sericulture development. To explore and reveal the molecular mechanisms of *S. ricini* in response to SariNPV infection, we employed RNA sequencing (RNA-seq), adopting isobaric tags for relative and absolute quantitation (iTRAQ), and carried out combination analysis of the obtained differentially expressed genes (DEGs) and proteins (DEPs). Through transcriptome sequencing, a total of 2535 DEGs were detected, and with iTRAQ, 434 DEPs with significant expression difference were identified. Through correlation analysis, we found that the expression trends of 116 differentially expressed proteins were the same as those of differentially expressed genes (including 106 up-regulated and 10 down-regulated). Twenty-five key differentially expressed genes (proteins) involved in several signaling and immune related pathways (mainly involving Toll, Imd, Jak-STAT and Wnt signaling pathways, as well as other immune related pathways) were screened through real-time quantitative PCR. Our results, not only provide insights into the pathogenic mechanism of SariNPV infection in ricin silkworm and the immune response mechanism within the host, but also provide a significant contribution for identifying and preventing diseases caused by SariNPV.

## 1. Introduction

*Samia ricini*, known as the eri-silkworm, is a multivoltine insect belonging to the lepidopteran family of Saturniidae. It has a relatively large body with a strong stress resistance and wide adaptability. The silk from *S. ricini* has the advantages of great elasticity, strong moisture absorption, and fine spinnability. SariNPV is a pathogen that commonly causes disease in *S. ricini* and severely threatens sericulture production. Its infection can easily cause significant economic loss to society. SariNPV belongs to the genus of baculovirus and has two virion phenotypes during its infectious life cycle: occlusion derived virus (ODV) and budded virus (BV). Of these, the ODV virus is encapsulated by polyhedrosis and primarily infects via oral administration. During infection, virus particles invade the peritrophic matrix of the midgut via oral infection and fall off through the interaction between the microvilli of intestinal epithelial cells and the envelope [1]. The spread of infection by SariNPV throughout the host is further enhanced by the spread of BVs to other tissues [2]. In the late stage of SariNPV infection in *S. ricini*, the link swells, the intersegment membrane becomes shiny, dark spots of different sizes appear on the back and two sides of the ventral valve, and, eventually, *S. ricini* dies. Though SariNPV infection in *S. ricini* has caused significant economic losses, little is known about the molecular mechanism of the interaction between SariNPV and *S. ricini*.

Changes in the expression levels of certain genes after NPV infects silkworm larvae, not only depend on their own phenotypic differences, but also are influenced by the physiological conditions of the host itself. It is a dynamic process between the pathogen and host. The immune barrier in the silkworm larvae activates the defense mechanism to prevent virus invasion, signals the immune pathway to trigger an immune response, and thus resists virus proliferation [3]. This change is reflected first at the genetic level. Studying the differences in the expression levels of host genes upon NPV infection can help in understanding the molecular mechanism of the host’s physiological response. Li et al. [4] obtain 5172 differentially expressed genes from midgut transcriptome analysis of *Antheraea pernyi* larvae infected by ApNPV, including heat shock protein (*comp45066_c0*, *comp42863_c0*), apoptosis-inducing factor (*comp63819_c0*), serine protease inhibitor (*comp32864_c0*, *comp39389_c0*), serine protease (*comp18400_c0*, *comp44655_c0*) and cytochrome P450 (*comp37472_c0*), etc. These differentially expressed genes are involved in several immune pathways, including Hippo signaling pathway, JAK-STAT signaling pathway, and others. In another study of BmNPV by Wang et al. [5], they found that the *Bmapaf-1* gene is differentially expressed after BmNPV infects *Bombyx mori*. These results show that *Bmapaf-1* is involved in anti-viral activities and resists the invasion of BmNPV by regulating the mitochondrial apoptosis pathway. Liu et al. also studied the expression levels of BmNPV-inhibiting proteins while analyzing the expression levels of BmNPV-related genes in silkworms, and found that *Bmlipase-1* and *serine protease II* (*BmSP-2*) are specifically expressed in the midgut of silkworms [6]. Furthermore, Guo et al. found that phosphoenolpyruvate carboxykinase (PEPCK) plays an important role in the process of gluconeogenesis in BmNPV infected cells, and BmPEPCK along with autophagy-related proteins inhibit the proliferation of BmNPV [7].

RNA-seq and iTRAQ are two widely and commonly used methods in entomological research. The advantages of these two methods are that they can be utilized to systematically analyze molecular regulatory networks and to study the systematic evolution of insects, which greatly advance the development of insect science. Previously, Jiang et al. used RNA-seq and iTRAQ technology to analyze the transcriptome and proteome of midgut tissue suffering from *A.* *pernyi* microsporidiosis, and disclosed functionally-related metabolic pathways and gene sets from the GO (gene ontology) and KEGG (Kyoto Encyclopedia of Genes and Genomes) annotation results. In combination with proteomic data, they further revealed the pathogenesis of *A.* *pernyi* microsporidiosis at the transcription level and at the protein level. 

Our current study is focused on exploring and revealing the molecular mechanisms of *S. ricini* infection by SariNPV by means of RNA-seq and iTRAQ technology. We expect to reveal the regulatory characteristics of SariNPV during *S. ricini* infection at the transcriptome and proteome levels, and discuss their biological functions during infection. The application of bioinformatics in analyzing immune genes (proteins) related to DEGs and DEPs will also lay a foundation for further exploration of the pathogenic molecular mechanism of SariNPV infection in *S. ricini* and in the immune response mechanism between the pathogen and host.

## 2. Materials and Methods

### 2.1. Sample Preparation

*Samia ricini* B7 variety (provided by the Sericultural Research Institute, Chinese Academy of Agricultural Sciences, Beijing, China), was fed with castor leaves until the 4th instar under the normal rearing conditions. The individuals of the same batch were divided into an experimental group and control group, with 30 individuals in each group. Three groups of repetitions were set up to facilitate the mortality statistics. The experimental group was fed with 7 μL SariNPV (containing 1.1 × 10^7^ PIB mL^−1^), while the control group was orally fed the same amount of 0.9% normal saline, and both groups were fed normally in the same environment. Midgut tissue was taken after 72 h as the experimental material. Three tubes were taken from each of the midguts in the experimental group and the control group, and were stored at −80 °C. The other individuals in the same group continued to be fed in the same sterile environment until obvious phenotype of the disease showed. Through microscopic examinations, we excluded the interference of bacteria, microbes, fungi, and other elements, so as to ensure that the phenotype traits were caused by SariNPV. All experiments carried out in three independent biological replicates, labeled, respectively, as the control group (MG-C1, MG-C2, MG-C3) and experimental group (MG-T1, MG-T2, MG-T3).

### 2.2. Transcripome Analysis

#### 2.2.1. RNA Extraction, cDNA Library Construction and Sequencing

RNAiso Plus (TaKaRa, Kusatsu, Japan) was used to extract the total RNA. ① RNA degradation and contamination was monitored on 1% agarose gels. ② RNA purity was checked using a NanoPhotometer^®^ spectrophotometer (IMPLEN, Munich, Germany). ③ RNA integrity was assessed using an RNA Nano 6000 Assay Kit from the Bioanalyzer 2100 system (Agilent Technologies, Santa Clara, CA, USA).

Sequencing libraries were generated using a NEBNext^®^ UltraTM RNA Library Prep Kit for Illumina^®^ (NEB, Ipswich, MA, USA) following the manufacturer’s recommendations, and index codes were added to attribute sequences to each sample. Briefly, mRNA was purified from total RNA using poly-T oligo-attached magnetic beads. Fragmentation was carried out using divalent cations under elevated temperature in NEBNext First Strand Synthesis Reaction Buffer (5×). First strand cDNA was synthesized using random hexamer primer and M-MuLV Reverse Transcriptase (RNase H−). Second strand cDNA synthesis was subsequently performed using DNA Polymerase I and RNase H. Remaining overhangs were converted into blunt ends via exonuclease/polymerase activities. After adenylation of the 3’ ends of DNA fragments, a NEB Next Adaptor with a hairpin loop structure was ligated to prepare for hybridization. In order to preferentially select cDNA fragments 250~300 bp in length, the library fragments were purified with AMPure XP system (Beckman Coulter, Beverly, MA, USA). Then, 3 µL USER Enzyme (NEB, Ipswich, MA, USA) was used with size-selected, adaptor-ligated cDNA at 37 °C for 15 min followed by 5 min at 95 °C before PCR. Then PCR was performed with Phusion High-Fidelity DNA polymerase, Universal PCR primers and Index (X) Primer. Finally, PCR products were purified (AMPure XP system) and library quality was assessed on the Agilent Bioanalyzer 2100 system (Agilent Technologies, Santa Clara, CA, USA).

The clustering of the index-coded samples was performed on a cBot Cluster Generation System using TruSeq PE Cluster Kit v3-cBot-HS (Illumia, Bologna, Italy) according to the manufacturer’s instructions. After cluster generation, library preparations were sequenced on an Illumina Novaseq platform and 150 bp paired-end reads were generated.

#### 2.2.2. Data Analysis and Quality Control

To ensure the quality and reliability of data analysis, the original data were filtered. This mainly included removing reads with adapters, removing reads containing N (N means that the base information cannot be determined), and removing low-quality reads (reads where the base number of Qphred ≤ 20 accounts for more than 50% of the entire read length). At the same time, the Q20, Q30 and GC contents of the clean data were calculated. All subsequent analyses were high-quality analyses based on clean data. We developed a high-quality de novo chromosome-level genome of *Samia ricini* B7 through the integration of PacBio long-read sequencing, Illumina short-read resequencing, and Hi-C sequencing (data not yet published). During the analyses, we also referred to an article on the genome of *Samia ricini* published by Lee et al. [8]. The reads mapped to each gene were calculated using the Counts feature (1.5.0-p3), and FPKM was calculated (sequenced kilobase pairs/million base pairs [9,10]) to estimate gene expression levels.

#### 2.2.3. Expression Analysis of Differential Expressed Genes

The DESeq2 software (1.16.1) was used to analyze the differential expression of the two groups, and the candidate genes were screened with reference to the differential gene detection method of Audic et al. [11]. The *p*-value of the test was adjusted by multiple hypothesis testing, and the false discovery rate (padj) ≤ 0.05 was used as a threshold screening method for DEGs to screen for genes with significant differences [12,13,14]. Then, for DEGs encoding proteins, GO and KEGG pathway enrichment analysis were performed.

### 2.3. Proteomics Methods

#### 2.3.1. Total Protein Extraction

The tissue sample was removed from the −80 °C refrigerator, ground into powder at a low temperature, and quickly transferred to a centrifuge tube that was pre-cooled with liquid nitrogen; an appropriate amount of protein lysis solution (100 mM ammonium bicarbonate, 8 M urea, 0.2% SDS, pH = 8) was then added, vortexed and mixed well, before sonication in an ice-water bath for 5 min to fully lyse. It was then centrifuged at 12,000× *g* at 4 °C for 15 min. The supernatant was taken and 10 mM of DTT red was added to the final concentration and allowed to react at 56 °C for 1 h. Then, enough IAM was added and reacted for 1 h at room temperature in the dark. Four times the volume of −20 °C pre-cooled acetone was added to precipitate at −20 °C for at least 2 h, centrifuged at 12,000× *g* for 15 min at 4 °C, and the precipitate was then collected. Then, 1 mL of −20 °C pre-cooled acetone was added to resuspend and wash the pellet, centrifuging at 12,000× *g* at 4 °C for 15 min, and it was then collected, air dried, and an appropriate amount of protein dissolving solution (6 M urea, 100 mM TEAB, pH = 8.5) was added to dissolve protein precipitation.

#### 2.3.2. Protein Testing

Using a Bradford protein quantification kit, BSA standard protein solution was prepared according to the instructions, with a concentration gradient ranging from 0 to 0.5 μg µL^−1^. Different concentration gradients of BSA standard protein solutions were taken and different dilutions of the sample solution were tested and then add to a 96-well plate to reach a volume of 20 µL, and this was repeated for each gradient 3 times. A volume of 180 µL was quickly added to G250 staining solution, left at room temperature for 5 min, and measured for absorbance at 595 nm. A standard curve with the absorbance of the standard protein solution was drawn and used calculate the protein concentration of the sample to be tested. Each 20 µg sample of proteins to be tested was subjected to 12% SDS-PAGE gel electrophoresis, in which the electrophoresis conditions of the concentrated gel were 80 V and 20 min, and the electrophoresis conditions of the separation gel were 120 V and 90 min. After the electrophoresis, it was stained with Coomassie Brilliant Blue R-250 and decolorized until the band was clear. Then iTRAQ labeling, fraction separation and liquid quality detection were carried out to generate mass detection raw data (raw).

#### 2.3.3. Identification and Quantification of Proteins

The search software Proteome Discoverer 2.2 (PD2.2, Thermo Fisher Scientific, Waltham, MA, USA) was used to search the result spectrum of each run according to the ricin silkworm protein database. The search parameters were set as follows: mass tolerance of precursor ions was 10 ppm and the mass tolerance of fragment ions was 0.02 Da. The immobilized modification was the alkylation modification of cysteine, the variable modification was methionine oxidation and iTRAQ tag modification, the N-terminal was acetylation modification and iTRAQ tag modification, allowing up to 2 missing cleavage sites. In order to improve the quality of analysis results, PD2.2 software was used for further filtering of the search results: spectral peptides (PSMs) with a credibility of more than 99% were credible PSMs, and proteins that contained at least one unique peptide (specific peptide) were credible for protein, only reliable peptides and proteins were retained, and FDR verification was performed to remove peptides and proteins with an FDR greater than 1%. The *t*-test was used to perform statistical analyses of the protein quantification results, and the proteins with significant quantitative differences between the experimental group and the control group were selected (when FC ≥ 1.2, *p* value ≤ 0.05, the up-regulated protein was selected, when FC ≤ 0.83, *p* value ≤ 0.05, down-regulated expression protein was screened) and defined as differentially expressed protein (DEP).

#### 2.3.4. Expression Analysis of Differential Proteins

Interproscan software was used for GO function annotation and KEGG was used for pathway analysis of the identified proteins [15]. For DEP, volcano map and cluster heat map analyses, as well as pathway enrichment analysis of GO and KEGG were performed [16].

### 2.4. qRT-PCR Verification

In order to verify the accuracy of the RNA-seq results, 10 DEGs, 5 up-regulated genes and 5 down-regulated genes, were randomly selected for qRT-PCR verification (Table S1). Meanwhile, 7 DEGs that were related to immune pathways were also selected and verify their expression by qRT-PCR (Table S2). The *Actin3* gene was used as the internal reference gene (F: CGGCTACTCGTTCACTACC, R: CCGTCGGGAAGTTCGTAAG), and Primer Premier 6.0 software was used to design gene-specific primers (synthesized by Shanghai Biological Engineering Co., Ltd., Shanghai, China).

RNAiso Plus (TaKaRa, Kusatsu, Japan) was used to extract total RNA. After treatment with DNase I (TaKaRa, Kusatsu, Japan), cDNA was synthesized using the M-MLV reverse transcriptase (RNase H-) kit (TaKaRa, Kusatsu, Japan) and diluted to 100 ng μL^−1^ as a template for qRT-PCR. The reaction was carried out in three biological replicates. After amplification, a melting curve was generated, and the data were analyzed with Light Cycle^®^96 software (Roche, Basel, Switzerland) and the relative expression level was calculated using the 2^−ΔΔCt^ method [17]. 

## 3. Results

### 3.1. Transcriptome Analysis of Midgut Samples

After screening the original data, six cDNA libraries were obtained to include 45,094,490 (96.30%), 44,600,114 (95.29%), 44,728,658 (94.63%), 44,402,340 (94.11%), 46,367,536 (95.76%), and 44,863,038 (93.92%) clean reads (Table 1). According to the fpkm values of all genes detected in each sample, the correlation coefficients of samples within and between groups were calculated and drawn as a heat map. The results show that the repeatability within samples is reliable and there are significant differences among samples at both mRNA level and protein level.

#### 3.1.1. Statistics and Identification of Differentially Expressed Genes

A total of 2535 DEGs were identified in the experimental group, of which 1264 were up-regulated DEGs and 1271 were down-regulated DEGs under screening difference criteria (Table 2; Figure S1). 

#### 3.1.2. Enrichment Analysis of DEGs

Based on the gene expression analysis, the DEGs were documented. Then, clusterProfiler software (http://bioconductor.org/packages/release/bioc/html/clusterProfiler.html, accessed on 7 February 2022) was used to perform GO function enrichment analysis and KEGG pathway enrichment analysis of the gene set (padj ≤ 0.05 was used as the threshold screening criterion for significant enrichment for both analyses).

1. Gene Ontology (GO) Function Annotation Analysis

With GO database annotation, a total of 2479 DEGs were annotated to 683 GO entries. Among them, 800 DEGs were annotated to 370 GO entries in the biological process group, 433 DEGs were annotated to 82 GO entries in the cellular component group, and 1246 DEGs were annotated to 231 GO entries in the molecular function group. The most significant 30 GO items in differentially expressed genes (10 most significant GO items for each ontology) involved in the biological process, cellular component, and molecular function (Table 3) were selected, and the results reflected the metabolic enrichment differences upon SariNPV infection in *S. ricini* at the gene expression level.

According to the significance of GO enrichment analysis, a scatter plot was drawn (Figure S2). In the biological process group, most DEGs were involved in various metabolic pathways including peptide metabolic process and cellular amide metabolic process, and in several biosynthesis processes, including organonitrogen compound biosynthetic process and cellular nitrogen compound biosynthetic process. In the cellular component group, most DEGs existed in the cytoplasm, ribosomes, protein-containing complexes or the mitochondria. In the molecular function group, the majority of DEGs were engaged in nucleotide binding, enzymatic activities, ATP interaction, etc. These results indicate that SariNPV infection in *S. ricini* may cause the denaturation of the midgut enzyme system and further affect protein synthesis and block metabolic pathways and biosynthetic processes in *S. ricini*.

2. KEGG function annotation analysis

Based on KEGG database annotations, 197 DEGs, including 193 up-regulated DEGs and 4 down-regulated DEGs, were found to be significantly enriched in 5 metabolic pathways. These metabolic pathways are related to mainly ribosome biogenesis in eukaryotes, oxidative phosphorylation, protein export, and proteasome (Table 4). The results show that SariNPV infection in *S. ricini* significantly affects nutrition metabolism and energy supply in *S. ricini*. Meanwhile, immune system and hormone secretion in *S. ricini* may also be affected.

#### 3.1.3. Verification of the Accuracy of Transcriptome Data by qRT-PCR

**Figure 1:**
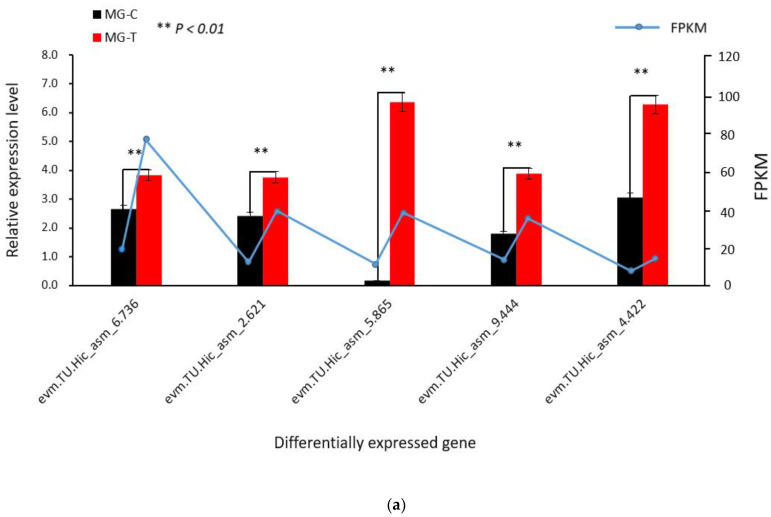

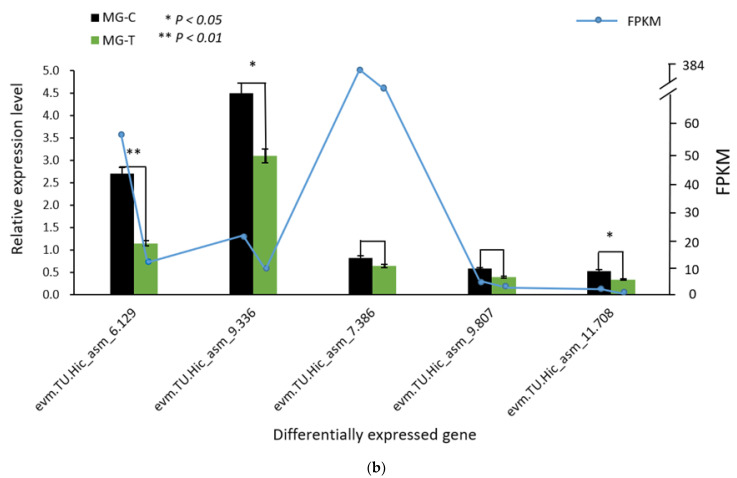
Ten DEGs verified by qRT-PCR: (**a**) up-regulated DEGs; (**b**) down-regulated DEGs. Note: The x axis is differentially expressed genes, the left y axis represents relative gene expression, and the right y axis represents FPKM value.

To verify the accuracy of the RNA-seq results, 10 DEGs were randomly selected for qRT-PCR verification using specific primers. These DEGs are mainly involved in metabolic processes, biosynthetic processes, enzyme activities, catalytic activities and molecular interactions. The qRT-PCR results show that the different expression trends of the validated genes are consistent with the transcriptome results. Among the 10 DEGs, *evm.TU.Hic_asm_6.736*, *evm.TU.Hic_asm_2.621*, evm.TU.Hic_asm_5.865, *evm.TU.Hic_asm_9.444*, *evm.TU.Hic_asm_4.422* showed an upward regulation trend, all the differences were extremely significant (Figure 1a), *evm.TU.Hic_asm_6.129*, *evm.TU.Hic_asm_9.336*, *evm.TU.Hic_asm_7.386*, *evm.TU.Hic_asm_9.807*, *evm.TU.Hic_asm_11.708* showed down-regulation trend, one of the differences was extremely remarkable, two were outstanding, and the remaining two were insignificant (Figure 1b).

### 3.2. Proteomic Analysis of Midgut Samples

In accordance to the screening and filtering criteria (Section 2.2.3), a total of 2971 differentially expressed proteins (DEPs) were identified.

#### 3.2.1. Protein Quantitative Analysis

Principal component analysis (PCA) results show that the two sample proteomes are significantly different (Figure S3). The analysis results of the repeatability coefficient of variation (CV) show that the curve of MG-T rises faster than that of MG-C, indicating that the overall sample repeatability is better (Figure S4).

#### 3.2.2. Differentially Expressed Protein Statistics

The ratio of the mean value of all biological replicate quantitative values of each protein in each group of samples was taken as the multiple of difference (FC). The relative quantitative value was tested by *t*-test, the significance of the difference was evaluated, and the corresponding *p*-value was calculated. According to the differential protein screening criteria (Section 2.2.3), a total of 2971 proteins were identified, among which, 762 were up-regulated DEPs, 198 were down-regulated DEPs (Table 5). The distribution of DEPs is shown in Figure S5.

#### 3.2.3. Enrichment Analysis of DEPs

1. GO enrichment analysis

A total of 313 DEPs were enriched in GO analysis (Table 6). In the biological process group, most DEPs were associated with the metabolic process. In the cellular component group, more than 50% of the DEPs were present in the organelles and nucleus. In the molecular function group, some DEPs interacted with nucleic acid and certain enzymes. This indicates that SariNPV infection may block metabolism and induce cell dysfunction and changes of enzymatic activities in *S. ricini*.

2. KEGG Pathway Enrichment Analysis

A total of 218 DEPs were found in the enrichment analysis of the KEGG pathway. The 20 most enriched KEGG pathways were selected according to the screening criteria, and a bubble chart of the enriched KEGG pathway was drawn (Figure S6). The results show that there are five DEPs in the signaling pathways that regulate pluripotency in stem cells, and account for about 55.56% of the pathways. However, in these pathways, the degree of DEP enrichment was highest. In this test, the p-value of the hypergeometric test of choline metabolism in cancer was the smallest. However, there was a big difference between the number of differentially expressed proteins in the pathways and the reliability of the test. Other related enrichment pathways were also associated with cancer, metabolism, biosynthesis and certain signaling pathways, which indicate that SariNPV infection in *S. ricini* may cause disease and serious damage to cell functions in *S. ricini*.

### 3.3. Association Analysis of Midgut Transcriptome and Proteome

#### 3.3.1. Transcriptome and Proteome Expression Regulation Analysis

**Figure 2:**
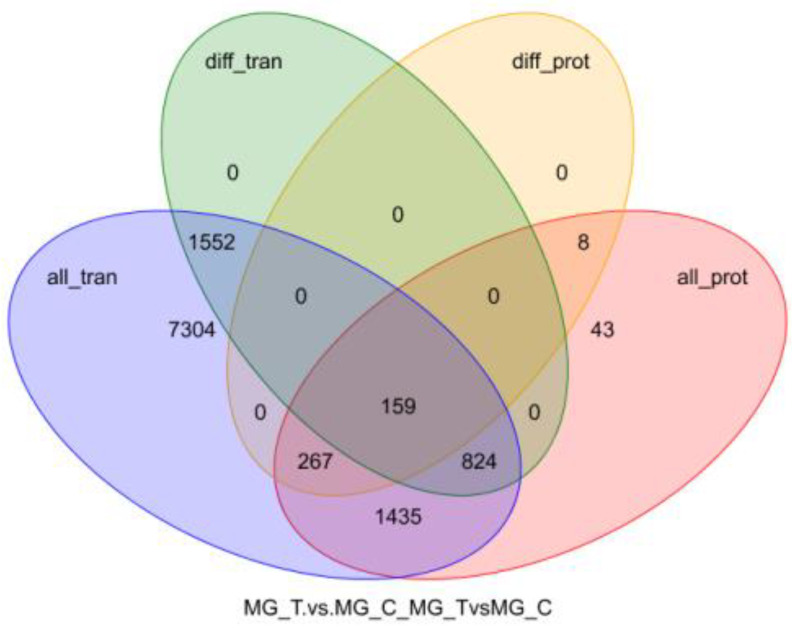
Venn diagram of transcriptome and proteome expression regulation.

**Figure 3:**
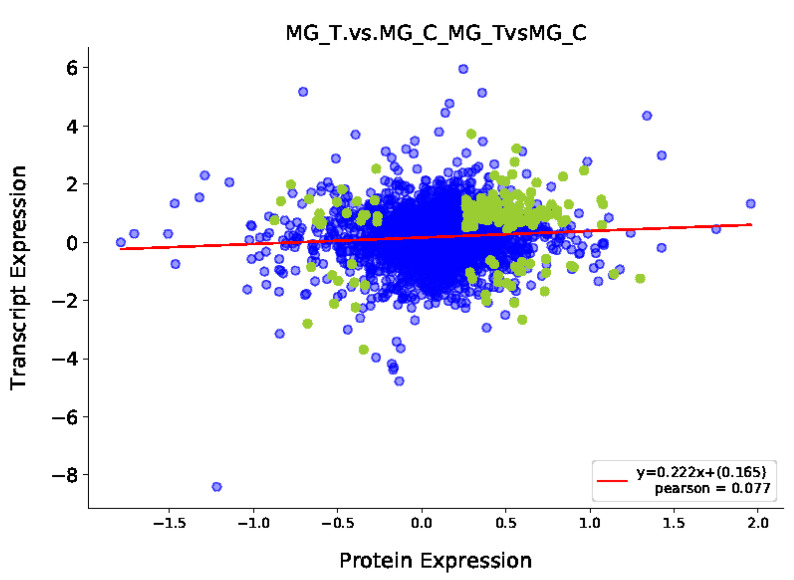
Correlation analysis of transcriptome and proteome expression. Note: Each dot represents a protein, green dots represent proteins with significantly different expression, and blue dots represent proteins with no significant difference in expression.

The mRNA information obtained from the transcriptome analysis was integrated with the protein information identified from the proteome analysis, and the corresponding relationship was found and drawn as a Venn diagram (Figure 2). Correlation analysis was performed on the multiples of difference between the two groups of genes (proteins) selected in the two omics (Figure 3). Results show that a total of 2535 DEGs and 434 DEPs were screened, of which 159 differentially expressed genes (proteins) were screened together.

#### 3.3.2. Joint Analysis of Transcriptome and Proteome Enrichment

Through integrating transcriptome and proteome data, we found and identified 290 annotated DEPs. Among them, 151 were involved in biological process, 106 were part of cellular components, and 219 had specific molecular functions, from a total of 476 (Table S3).

**Figure 4:**
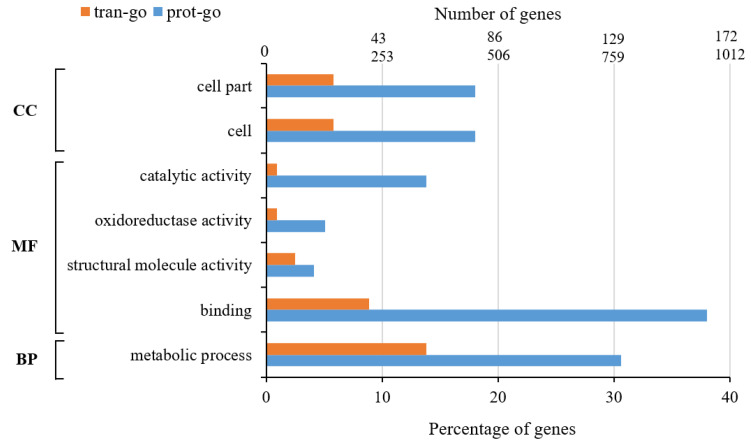
Correlation bar graph of DEGs/DEPs in GO enrichment of transcriptome and proteome.

In GO functional enrichment analysis, by selecting genes (proteins) that have common significant differences in the transcriptome and proteome, we created a histogram, as shown in Figure 4. It has been found that the differentially expressed genes (proteins) jointly affect the metabolic process, the cellular component and cell parts, catalytic activity, oxidoreductase activity, structural molecular activities and molecular interactions in cells.

Among them, the results of cells and cell parts were the same, each with 78 DEPs and 148 DEGs, accounting for 18.0% of the total DEPs and 5.8% of the total DEGs. Sixty DEPs were in the catalytic activity group, accounting for the total difference 13.8% of the DEPs. Twenty-two DEGs, accounting for 0.9% of the total DEGs, were in the branch of catalytic activity. There were 22 DEPs and 22 DEGs in the oxidoreductase activity group. A total of 18 DEPs and 63 DEGs were in the structural molecular activity group, accounting for 4.1% of the total DEPs and 2.5% of the total DEGs, respectively. The number of DEPs in molecular binding group was the largest, with 165 DEPs, accounting for 38% of the total DEPs and 225 DEGs were in the molecular binding group, accounting for 8.9% of the total DEGs. A total of 133 DEPs were in the metabolic process group, accounting for 30.6% of the total DEPs, however, the number of DEGs in the metabolic process group was the largest, reaching 350, accounting for 13.8% of the total DEGs. Furthermore, there were other GO functions that were also involved, such as transportation activities, cell component organization, biogenesis, and cellular localization.

### 3.4. Verification of Differentially Expressed Genes in the Immune Pathway by qRT-PCR

**Figure 5:**
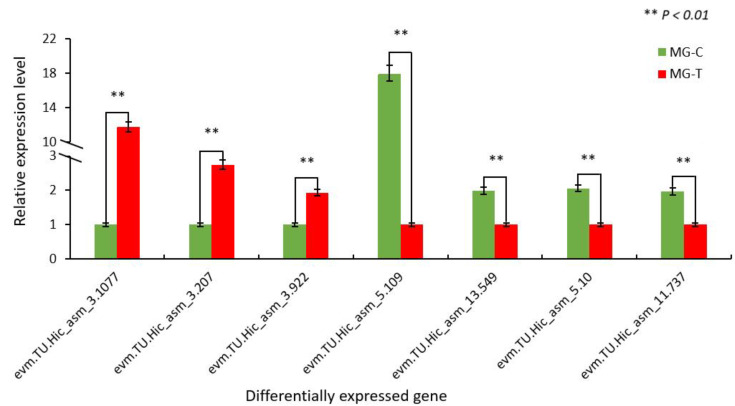
qRT-PCR verification of differentially expressed genes in the immune pathway.

Specific primers designed for seven DEGs designed were utilized for qRT-PCR analysis. The qRT-PCR results show different expression trends in the validated genes. Among the seven DEGs, *evm.TU.Hic asm_3.1077*, *evm.TU.Hic_asm_3.207*, and *evm.TU.Hic_asm_3.922* showed an upward trend, however, *evm.TU.Hic_asm_5.109*, *evm.TU.Hic_asm_13.549*, *evm.TU.Hic_asm_5.10* and *evm.TU.Hic_asm_11.737* showed a downward trend. The seven DEGs showed remarkable differences, which are also consistent with the results obtained from Illumina sequencing data (Figure 5).

## 4. Discussion

Baculovirus has only been isolated from arthropods (mostly from insects) so far, and all of them have a relatively narrow range of hosts. When baculovirus infects non-susceptible insects or insect cell lines, the virus replication cycle can be blocked in one or several links, but this blockade has specificity towards the host’s ethnicity [18]. Studies have shown that viral gene expression and DNA replication are key steps in determining host specificity [19,20]. The gene expression of nucleopolyhedrovirus is consisted of four stages: immediate early gene expression (α-phase), early gene expression (β-phase), late gene expression (γ-phase) and extremely late gene expression (δ-phase) [21]. Relevant studies in silkworms have shown that the expression of baculovirus genes and the formation of virus particles follow an orderly cascade model. In the cascade regulation model, each stage is dependent on the product of gene expression from the prior stage. The transcription of early genes regulates the replication of viral DNA, while the expression of late viral genes depends on the expression of early genes and DNA replication [22,23]. Virus proliferation often induces various physiological and metabolic changes in the host. These changes are resulted from virus infection and host immunity. Meanwhile, the host organism’s defense against virus infection is triggered. Eventually, the virus can successfully invade the host through multiple lines in the defense stages including nucleic acid replication, viral protein synthesis, viral particle assembly and morphogenesis.

Through the correlation analysis of DEGs and DEPs identified by transcriptome sequencing and proteome quantification, a total of 2535 DEGs and 434 DEPs were screened, in which 159 differentially expressed genes (proteins) were screened together. These differentially expressed genes (proteins) provide potential insights into elaborating the complex molecular mechanism of SariNPV infection in S. *ricini*. These differentially expressed genes (proteins) also regulate the Toll and Imd signaling pathways, Jak-STAT signaling pathway, Wnt signaling pathway, cytochrome P450 and other immune-related pathways. Together the host constitutes a complex pathological response upon SariNPV infection in *S. ricini*.

**Figure 6:**
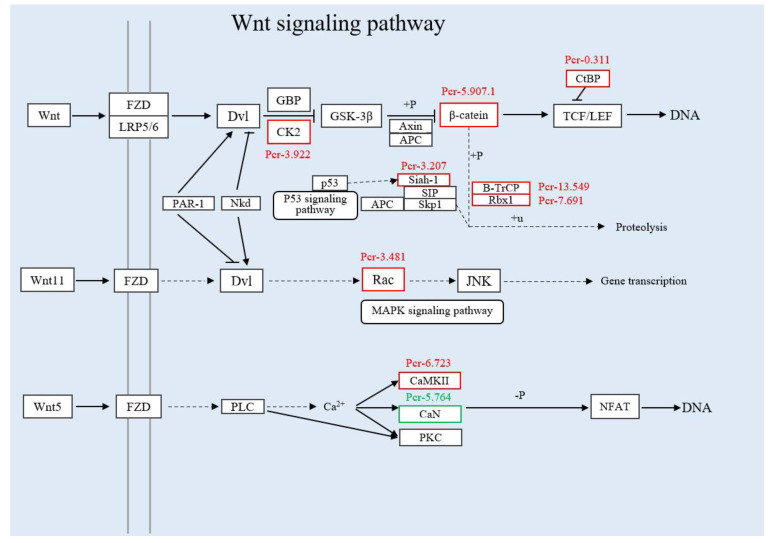
DEPs involved in the Wnt signaling pathway. The two vertical gray lines on the left represent the cell membrane, the solid line is the activation effect, and the dotted line is the indirect effect.

The Wnt signaling pathway is a highly conserved signaling pathway in the perspective of species evolution. This signaling pathway is very similar from *Drosophila* to humans [24]. The WNT protein encoded by the *Wnt* gene binds to receptors on the cell membrane through autocrine or paracrine action. The WNT protein activates various intracellular signals, signals transduction molecules, transmits growth stimulating signals, regulates the expression of target genes, participates in different developmental mechanisms, and determines cell destiny. In this study, we identified 8 up-regulated expression proteins (Pcr-3.481, Pcr-3.207, Pcr-0.311, Pcr-5.907.1, Pcr-7.691, Pcr-3.922, Pcr-13.549, and Pcr-6.723) and 1 down-regulated expression protein (Pcr-5.764). Due to virus invasion, the receptor FZD (Frizzled) in complex with WNT protein transfers the signal to the cell and activates DVL, which further stimulates the up-regulation of protein CK2, thereby inhibiting serine-threonine kinase (GSK3β) (Figure 6). The tumor suppressor protein Axin and APC are used as the backbone to inhibit β-catenin controlled by the up-regulated protein Pcr-5.907.1, and meanwhile, together with the CtBP controlled by the up-regulated protein Pcr-0.311 to affect the transcription factor TCF/LEF. This process leads to the inhibition of downstream gene expression, which affects cell mitosis. On the other hand, B-TrCP, Rbxl, Siah-l related proteins Pcr-13.549, Pcr-7.691, Pcr-3.207 are all up-regulated, and indirectly act in the phosphorylation process of β-catenin, and final ubiquitination leads to protein degradation. The activated FZD can further activate DVL and then activate the small G protein Rac, which makes the Pcr-3.481 protein up-regulated and participate in the Wnt/PCP signal transduction process. Eventually this leads to the regulation of the actin cytoskeleton, cell adhesion and gene transcription, and activation of the MAPK signaling pathway. Since the FZD receptor is a G protein-coupled receptor, after the binding of FZD to WNT protein, Wnt signaling pathway is activated. The phospholipase C (PLC) located on the plasma membrane is further activated by the G protein, leading to an increase in the concentration of the signal molecule Ca^2+^ and to the up-regulation of the expression of the Pcr-6.723 protein. Pcr-5.764 down-regulates the expression, and then activates calmodulin-dependent protein kinase II (CaMKII) and Calcineurin (CaN). CaN can activate T cell-related cytoplasmic protein nuclear factor (NFAT) through dephosphorylation to promote the expression of several genes in cardiac and skeletal muscle cells, as well as the expression of pro-inflammatory genes in lymphocytes. The identification of DEPs by detecting the distribution of their protein expression levels also indicates that multiple cancer-related pathways were involved. Our results show that the expression trend of differentially expressed proteins involved in the Wnt signaling pathway is highly up-regulated, which indicates that SariNPV is stimulated after infecting *S. ricini*. In addition, the encoded proteins are expressed to varying degrees, which leads to the activation of the Wnt signaling pathway and resistance to the invasion and the proliferation of pathogens in cells.

**Figure 7:**
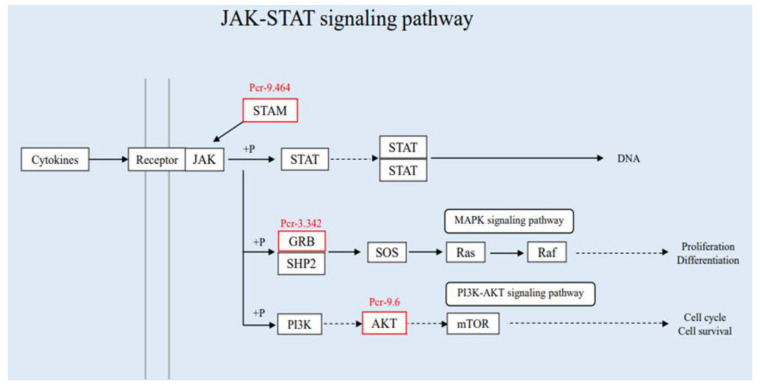
DEPs involved in the JAK-STAT signaling pathway. The two vertical gray lines on the left represent the cell membrane, the solid line is the activation effect, and the dotted line is the indirect effect.

The Janus kinase/signal transducers and activators of transcription (JAK-STAT) signal pathway is a recently discovered principal signaling transduction pathway for a variety of cytokines and growth factors [25,26,27]. JAK activation stimulates many important biological processes including cell proliferation, differentiation, apoptosis, and immune regulation. Chemical signals outside the cell are transmitted cross the cell membrane and the information is delivered to the gene promoter on the DNA within the cell nucleus, which leads to changes in the levels of DNA transcription and activity [28]. JAK and STAT are two key components in the signaling pathways that regulate cell growth, differentiation, survival, and pathogen resistance [29]. The activated JAKs subsequently phosphorylate targets including STATs and this further activates downstream pathways. Therefore, as an inflammatory signaling pathway for stress, this cascade must respond quickly [30]. Once pathogens invade, JAK-STAT cascade is immediately activated and assembled to the nucleus to perform transcriptional functions. In addition, JAK-STAT can be considered as an antiviral and antiproliferative interferon, such as IL-6. The JAK-STAT signaling pathway in silkworm is evolutionarily similar to that in *Drosophila* [31]. Upon BmNPV infection in silkworm, the up-regulated expression of *BmStat* gene in the midgut can be detected, which indicates that the JAK-STAT signaling pathway is activated and participates in the antiviral immune response [32]. In this study, three differentially expressed proteins (Pcr-9.464, Pcr-3.342, Pcr-9.6) related to the JAK-STAT signaling pathway were screened from the obtained DEPs. The three differentially expressed proteins related to the JAK-STAT signaling pathway all showed an up-regulation trend (Figure 7). This indicates that after SariNPV infects *S. ricini*, the pathogen invades the host body, and activates the JAK-STAT signaling pathway to participate in the immune response and resist virus invasion. 

**Figure 8:**
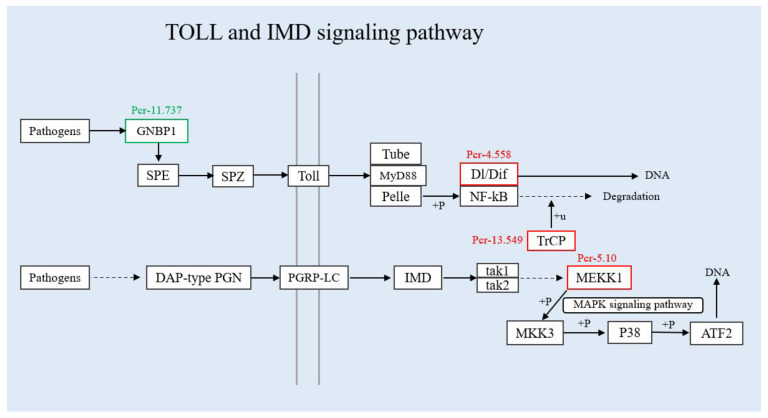
DEPs involved in the TOLL and IMD signaling pathways. The two vertical gray lines on the left represent the cell membrane, the solid line is the activation effect, and the dotted line is the indirect effect.

Toll and Imd signaling pathways are the innate immune pathways that can exist independently but cooperate with each other [33,34]. There are about 10 copies of Toll receptors (TLRs in mammals and insects). All mammalian TLRs participate in the innate immune response, while only one Toll receptor in insects has immune function [35]. Toll-like receptors (TLRs) are also pattern recognition receptors (PRRs) and can directly recognize foreign substances and activate signal transduction responses. Toll receptors in insects have no PRR function and must be in complex with its partner Spätzle protein. The signal transduction process can only be started when TLRs are in complex with Spätzle protein. Once the Toll–Spätzle complex is formed, the signal transduction process is turned on, which induces conformational changes in Toll receptors [36]. This further triggers the activation of Dl (Dorsal)/Dif (Dorsal-related immunity factor) proteins, but inhibits protein dissociation with the ankyrin of the IkB (inhibitor of nuclear factor kappa B) homologue. Then NF-kB (nuclear factor kappa B) is activated and downstream effector genes are transcribed [37]. Toll receptors trigger the immune response against pathogens via recognizing PAPMs and harmful endogenous substances. The activation of TLRs can stimulate a strong immune response, which benefits the host from resisting pathogen infection and avoiding tissue damage. However, excessive immune responses can also bring adverse effects, such as endotoxic shock and autoimmune diseases. Our current study screened three up-regulated proteins (Pcr-4.558, Pcr-5.10, Pcr-13.549) and one down-regulated protein (Pcr-11.737) (Figure 8). After SariNPV infects *S. ricini*, the expression levels of DEGs and DEPs in the Toll and Imd signaling pathways indicate that the Toll and Imd signaling pathways trigger a defense mechanism against the invasion of SariNPV, which plays a vital role in the immune response.

CYP450s (Cytochromes P450) are a superfamily of enzymes containing the factor heme, and oxidizing various endogenous and exogenous compounds [38]. When foreign pathogens invade the host body, CYP450 begins to oxidize and metabolize its substrates. Regarding CYP450’s metabolisms of action, there are two pathways known as metabolic detoxification and metabolic activation. The products from metabolic activation usually have strong toxicities and even have carcinogenic effects. In this study, 9 DEPs were identified, in which 3 were up-regulated (Pcr-1.120, Pcr-1.119, and Pcr-1.117), and 1 was down-regulated (Pcr-3.1077), 5 were multi-regulated proteins (Pcr-9.598, Pcr-2.393, Pcr-5.455, Pcr-5.449, and Pcr-11.135). Among them, 4 (Pcr-9.598, Pcr-2.393, Pcr-5.455, and Pcr-5.449) were up-regulated expression proteins, but Pcr-11.135 was a down-regulated expression protein, which collectively represents glutathione transferase. According to the KEGG pathway analysis, the screened DEPs act on a series of chemical molecules including 1,2-dihydroxy-naphthalene and trichloroethanol-glucuronide, and then affect the biosynthesis of steroid hormones. The results of the different expression levels of related gene proteins in the cytochrome P450 signaling pathway upon SariNPV infection in *S. ricini* indicate that they are involved in the body’s immune response.

## 5. Conclusions

**Figure 9:**
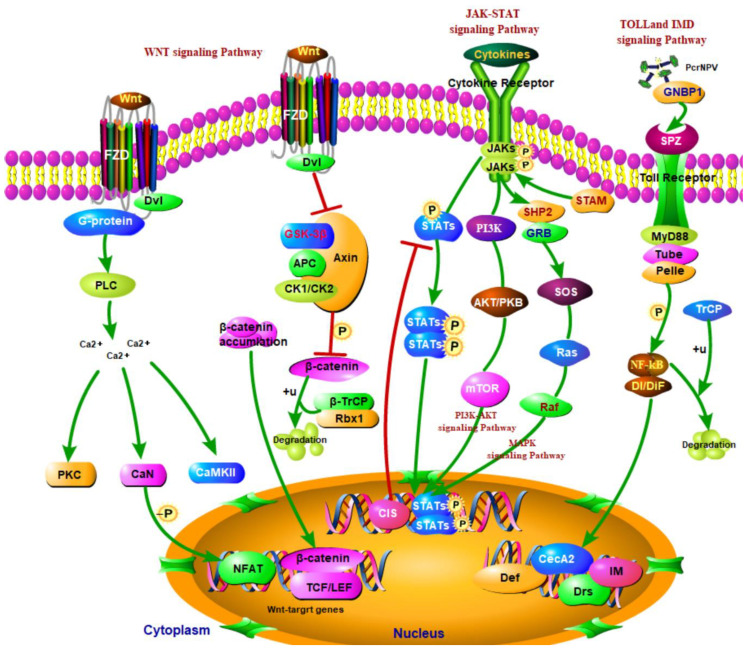
Immune pathways involved in *S. ricini* after SariNPV infection.

The pathways discussed identified in this present study, such as the Wnt signaling pathway, JAK-STAT signaling pathway, MAPK signaling pathway, calcium signaling pathway, and Hippo signaling pathway, are involved in the immune responses in host upon SariNPV infection, to resist the invasion of pathogens. In the perspective of metabolism, other pathways such as MAPK signaling pathway, oxidative phosphorylation and glucagon signaling pathway provide energy for immune responses against pathogen invasion, and positively regulate the signal transduction pathway by supplying cellular ATP. This shows that once SariNPV infects the *S. ricini*, the midgut tissue initiates immune defense. The immune response of these important immune pathways to resist virus infection to the induction of SariNPV, also proves a series of complex physiological and pathological changes caused by the related DEGs and DEPs after virus invasion (Figure 9). In conclusion, this study is of great significance in providing genetic insights into the molecular mechanism of pathogen-host interaction in SariNPV-infected silkworm, and in exploring the immune response mechanism and network in *S. ricini* infected with SariNPV.

## Figures and Tables

**Table 1:** Statistical analysis of the transcriptome sequence data.

Sample	Raw-Reads	Clean-Reads	Clean-Bases	Error-Rate	Q20 (%)	Q30 (%)	GC-Pct
MG-C1	46,825,600	45,094,490	6.76G	0.03%	98.09	93.93	45.52%
MG-C2	46,803,912	44,600,114	6.69G	0.03%	98.08	93.93	45.61%
MG-C3	47,270,958	44,728,658	6.71G	0.03%	97.63	92.89	45.68%
MG-T1	47,180,212	44,402,340	6.66G	0.02%	98.13	94.12	45.17%
MG-T2	48,422,268	46,367,536	6.96G	0.03%	96.49	90.81	44.96%
MG-T3	47,769,512	44,863,038	6.73G	0.02%	98.26	94.29	43.20%

**Table 2:** Statistical analysis of differentially expressed genes.

DEGs	All	Up	Down	Threshold		
MG-T vs. MG-C	2535	1264	1271	DESeq2	padj ≤ 0.05	|log2(FC)| ≥ 1.5

**Table 3:** GO function classification of differentially expressed genes.

GO Classification	GO Function	Number of Unigenes	Up-Regulated	Down-Regulated
Biological Process	translation	78	77	1
	peptide metabolic process	80	79	1
	peptide biosynthetic process	78	77	1
	amide biosynthetic process	78	77	1
	organonitrogen compound biosynthetic process	109	103	6
	macromolecule biosynthetic process	168	118	50
	gene expression	170	119	51
	cellular nitrogen compound biosynthetic process	165	115	50
	cellular amide metabolic process	81	79	2
	cellular macromolecule biosynthetic process	167	118	47
Cellular Component	cytoplasmic part	114	106	8
	ribonucleoprotein complex	68	68	0
	ribosome	58	58	0
	cytoplasm	127	115	12
	protein-containing complex	144	112	32
	non-membrane-bounded organelle	77	63	14
	intracellular non-membrane-bounded organelle	77	63	14
	mitochondrion	29	27	2
	mitochondrial part	21	19	2
	peptidase complex	15	14	1
Molecular Function	structural constituent of ribosome	55	55	0
	structural molecule activity	63	59	4
	ATP binding	150	69	81
	adenyl ribonucleotide binding	150	69	81
	adenyl nucleotide binding	150	69	81
	cytoskeletal protein binding	26	6	20
	threonine-type endopeptidase activity	11	11	0
	threonine-type peptidase activity	11	11	0
	RNA-binding	46	38	8
	microtubule binding	13	4	9

**Table 4:** KEGG function classification of differentially expressed genes.

KEGG ID	Pathway	No. of Unigenes	Up-Regulated	Down-Regulated	Padj
bmor03010	Ribosome	72	72	0	3.67 × 10^−13^
bmor03008	Ribosome biogenesis in eukaryotes	39	36	3	1.10 × 10^−6^
bmor00190	Oxidative phosphorylation	49	49	0	1.45 × 10^−5^
bmor03060	Protein export	15	14	1	0.000124708
bmor03050	Proteasome	22	22	0	0.002445682

**Table 5:** DEPs statistics.

Samples	Number of Co-Identified Proteins	Regulated Type	FC > 1.2	FC > 1.3	FC > 1.5	FC > 2.0
MG-T vs. MG-C	2971	up-regulated	360	264	118	20
		down-regulated	102	66	27	3

**Table 6:** Significant enrichment of GO of DEPs of (MG-T vs. MG-C).

GO Classification	GO Term	x/n		Up	Down	*p*-Value	GO ID
Biological Process	cellular macromolecule metabolic process	67/313	21.41%	60	7	0.030463	GO:0044260
	cellular nitrogen compound metabolic process	50/313	15.97%	41	9	0.045171	GO:0034641
	nucleobase-containing compound metabolic process	31/313	9.90%	27	4	0.046252	GO:0006139
	ribonucleoprotein complex biogenesis	6/313	1.92%	6	0	0.048092	GO:0022613
Cellular Component	intracellular membrane-bounded organelle	43/313	13.74%	36	7	0.002168	GO:0043231
	nucleolus	23/313	7.35%	21	2	0.009377	GO:0005634
	organelle	59/313	18.85%	48	11	0.025455	GO:0043226
	intracellular organelle	58/313	18.53%	48	10	0.036687	GO:0043229
Molecular Function	nucleic acid binding	48/313	15.34%	42	6	0.010857	GO:0003676
	glucosidase activity	2/313	0.64%	2	0	0.021454	GO:0015926
	Ran GTPase binding	3/313	0.96%	3	0	0.024863	GO:0008536

Note: x/n: the number of differential proteins annotated for this GO entry/the number of differential proteins annotated by all GO, up: up-regulate DEPs, down: down-regulate DEPs.

## Data Availability

The data presented in this study are available in article or supplementary materials.

## Supplementary Materials

The following supporting information can be downloaded at: https://www.mdpi.com/article/10.3390/insects13030294/s1, Figure S1: Volcano map of the DEGs; Figure S2: Scatter plot of GO enrichment analysis; Figure S3: Repeatable CV analysis; Figure S4: PCA analysis; Figure S5: Volcano map of the DEPs; Figure S6: Scatter plot of DEPs’ KEGG pathway analysis; Table S1: 10 Primer sequences of DEGs for qRT-PCR verification; Table S2: 7 Primer sequences of DEGs for qRT-PCR verification; Table S3: Transcriptome and proteome GO enrichment DEGs/DEPs statistics table.

## References

[1] Zhu S., Wang W., Wang Y., Yuan M., Yang K. (2013). The baculovirus core gene *ac83* is required for nucleocapsid assembly and *per*
*os* infectivity of *Autographa californica nucleopolyhedrovirus*. J. Virol..

[2] Zhu S.M., Li H. (2016). Research advances in baculovirus occlusion-derived virions. Bing Du Xue Bao.

[3] Hillyer J.F. (2016). Insect immunology and hematopoiesis. Dev. Comp. Immunol..

[4] Li X.S., Wang G.B., Sun Y., Wei L., He Y.Z., Wang F.C., Jiang Y.R., Qin L., Joe H.J. (2016). Transcriptome analysis of the midgut of the Chinese oak silkworm *Antheraea pernyi* infected with *Antheraea pernyi* nucleopolyhedrovirus. PLoS ONE.

[5] Wang X.Y., Ding X.Y., Chen Q.Y., Zhang K.X., Li M.W. (2020). *Bmapaf-1* is involved in the response against BmNPV Infection by the mitochondrial apoptosis pathway. Insects.

[6] Liu Y., Ai J.W., Tang Y., Xue H., He X.J., Zheng Y. (2019). Expression analysis of antiviral genes related to BmNPV in silkworm(*Bombyx mori*) and screening of practical molecular markers. J. Agric. Biotechnol..

[7] Guo H., Xu G., Wang B.B., Xia F., Sun Q., Wang Y.M., Xie E.Y., Lu Z.Y., Jiang L., Xia Q.Y. (2019). Phosphoenolpyruvate carboxykinase is involved in antiviral immunity against *Bombyx mori* nucleopolyhedrovirus. Dev. Comp. Immunol..

[8] Lee J., Nishiyama T., Shigenobu S., Yamaguchi K., Suzuki Y., Shimada T., Katsuma S., Kiuchi T. (2021). The genome sequence of *Samia ricini*, a new model species of lepidopteran insect. Mol. Ecol. Resour..

[9] Dewey C.N., Li B. (2011). RSEM: Accurate transcript quantification from RNA-Seq data with or without a reference genome. BMC Bioinform..

[10] Trapnell C., Williams B.A., Pertea G., Mortazavi A., Kwan G., Baren M., Salzberg S.L., Wold B.J., Pachter L. (2010). Transcript assembly and quantification by RNA-Seq reveals unannotated transcripts and isoform switching during cell differentiation. Nat. Biotechnol..

[11] Samarskiĭ A., Claverie J.M. (1997). The significance of digital gene expression profiles. Genome Res..

[12] Anders S., Huber W. (2010). Differential expression analysis for sequence count data. Genome Biol..

[13] Love M.I., Huber W., Anders S. (2014). Moderated estimation of fold change and dispersion for RNA-seq data with DESeq2. Genome Biol..

[14] Smyth G.K. (2010). edgeR: A Bioconductor package for differential expression analysis of digital gene expression data. Bioinformatics.

[15] Philip J., David B., Chang H.Y., Matthew F., Li W., Craig M.A., Hamish M.W., John M., Alex M., Gift N. (2014). InterProScan 5: Genome-scale protein function classification. Bioinformatics.

[16] Wei H.D., Sherman B.T., Lempicki R.A. (2009). Bioinformatics enrichment tools: Paths toward the comprehensive functional analysis of large gene lists. Nucleic Acids Res..

[17] Livak K.J., Schmittgen T. (2001). Analysis of relative gene expression data using real-time quantitative PCR and the 2-ΔΔ Ct method. Methods.

[18] Kurasawa J.H., Park A., Sowers C.R., Halpin R.A., Ikeda Y. (2020). Chemically defined, high-density insect cell-based expression system for scalable AAV vector production. Mol. Ther. Methods Clin. Dev..

[19] Wang X.Y., Yu H.Z., Xu J.P., Zhang S.Z., Yu D., Liu M.H., Wang L.L. (2017). Comparative subcellular proteomics analysis of susceptible and near-isogenic resistant *Bombyx mori* (Lepidoptera) larval midgut response to BmNPV infection. Sci. Rep..

[20] Zhang X., Guo R., Kumar D., Ma H., Liu J., Hu X., Cao G., Xue R., Gong C. (2016). Identification, gene expression and immune function of the novel *Bm-STAT* gene in virus-infected *Bombyx mori*. Gene.

[21] Vanarsdall A., Mikhailov V., Rohrmann G. (2007). Baculovirus DNA replication and processing. Curr. Drug Targets.

[22] Oomens A., Blissard G.W. (1999). Requirement for GP64 to drive efficient budding of autographa californica multicapsid nucleopolyhedrovirus. Virology.

[23] Dong X.L., Liu T.H., Wang W., Pan C.X., Pan M.H. (2015). *BmREEPa* Is a novel gene that facilitates BmNPV entry into silkworm cells. PLoS ONE.

[24] Morgan R., Ankrah R., El-Tanani S., Loadman P.M., Pattterson L., Rudland P.S., El-Tanani M. (2017). Wnt signaling as a therapeutic target in cancer and metastasis. Introduction to Cancer Metastasis.

[25] Carlota R., Ainhoa O., Iolanda L., Beriat M., Jesus E. (2014). Suppressor of cytokine signaling 1-derived peptide inhibits Janus kinase/signal transducers and activators of transcription pathway and improves inflammation and atherosclerosis in diabetic mice. Arterioscler. Thromb. Vasc. Biol..

[26] Boudny V., Kovarik J. (2002). JAK/STAT signaling pathways and cancer Janus kinases/signal transducers and activators of transcription. Neoplasma.

[27] Zhou X., Sun Y., Gao J., Sun Y., Liu C., Wang L. (2018). Immune function of a Rab-related protein by modulating the JAK-STAT signaling pathway in the silkworm, *Bombyx mori*. Arch. Insect Biochem. Physiol..

[28] Yang Q.Q., Tan H., Fu Z.P., Ma Q., Song J.L. (2017). HSP90 inhibitor 17-AAG plays an important role in JAK3/STAT5 signaling pathways in HTLV-1 infection cell line HUT-102. Zhonghua Xue Ye Xue Za Zhi.

[29] Rawlings J.S. (2004). The JAK/STAT signaling pathway. J. Cell Sci..

[30] Ren F., Wang B., Yue T., Yun E.Y., Ip Y.T., Jiang J. (2010). Hippo signaling regulates drosophila intestine stem cell proliferation through multiple pathways. Proc. Natl. Acad. Sci. USA.

[31] Li J., Fan X., Li W.X. (2003). Coactivation of STAT and Ras is required for germ cell proliferation and invasive migration in *Drosophila*. Dev. Cell.

[32] Liu W.L., Liu J.B., Lu Y.H., Gong Y.C., Zhu M., Chen F., Liang Z., Zhu L.K., Su L., Hu X.L. (2015). Immune signaling pathways activated in response to different pathogenic micro-organisms in *Bombyx mori*. Mol. Immunol..

[33] Gregorio D.E., Spellman P.T., Tzou P., Rubin G.M., Lemaitre B. (2002). The Toll and Imd pathways are the major regulators of the immune response in *Drosophila*. EMBO J..

[34] Han M., Qin S., Song X., Li Y., Jin P. (2013). Evolutionary rate patterns of genes involved in the *Drosophila* Toll and Imd signaling pathway. BMC Evol. Biol..

[35] Imler J.L., Zheng L.B. (2004). Biology of Toll receptors: Lessons from insects and mammals. J. Leukoc. Biol..

[36] Tanji T., Ip Y.T. (2005). Regulators of the Toll and Imd pathways in the *Drosophila* innate immune response. Trends Immunol..

[37] Takeda K., Kaisho T., Akira S. (2003). Toll-like receptors. Annu. Rev. Immunol..

[38] Lamb D.C., Follmer A.H., Goldstone J.V., Nelson D.R., Warrilow A.G., Price C.L., True M.Y., Kelly S.L., Poulos T.L., Stegeman J.J. (2019). On the occurrence of cytochrome P450 in viruses. Proc. Natl. Acad. Sci. USA.

