# Supplementary material for: Integration of Transcriptomic and Proteomic Analyses Reveals New Insights into the Regulation of Immune Pathways in Midgut of Samia ricini upon SariNPV Infection
Source: Insects. 2022 Mar 16;13(3):294. doi: 10.3390/insects13030294 (PMC8949121; doi:10.3390/insects13030294)
Supplement: Supplementary file 1 [file insects-13-00294-s001.zip › insects-1611401-supplementary.pdf]

Supplementary Materials

Figure S1: Volcano map of the DEGs

Figure S2: Scatter plot of GO enrichment analysis

Figure S3: Repeatable CV analysis

Figure S4: PCA analysis

Figure S5: Volcano map of the DEPs

Figure S6: Scatter plot of DEPs' KEGG pathway analysis

Table S1: 10 Primer sequences of DEGs for qRT-PCR verification

Table S2: 7 Primer sequences of DEGs for qRT-PCR verification

Table S3: Transcriptome and proteome GO enrichment DEGs/DEPs statistics table.

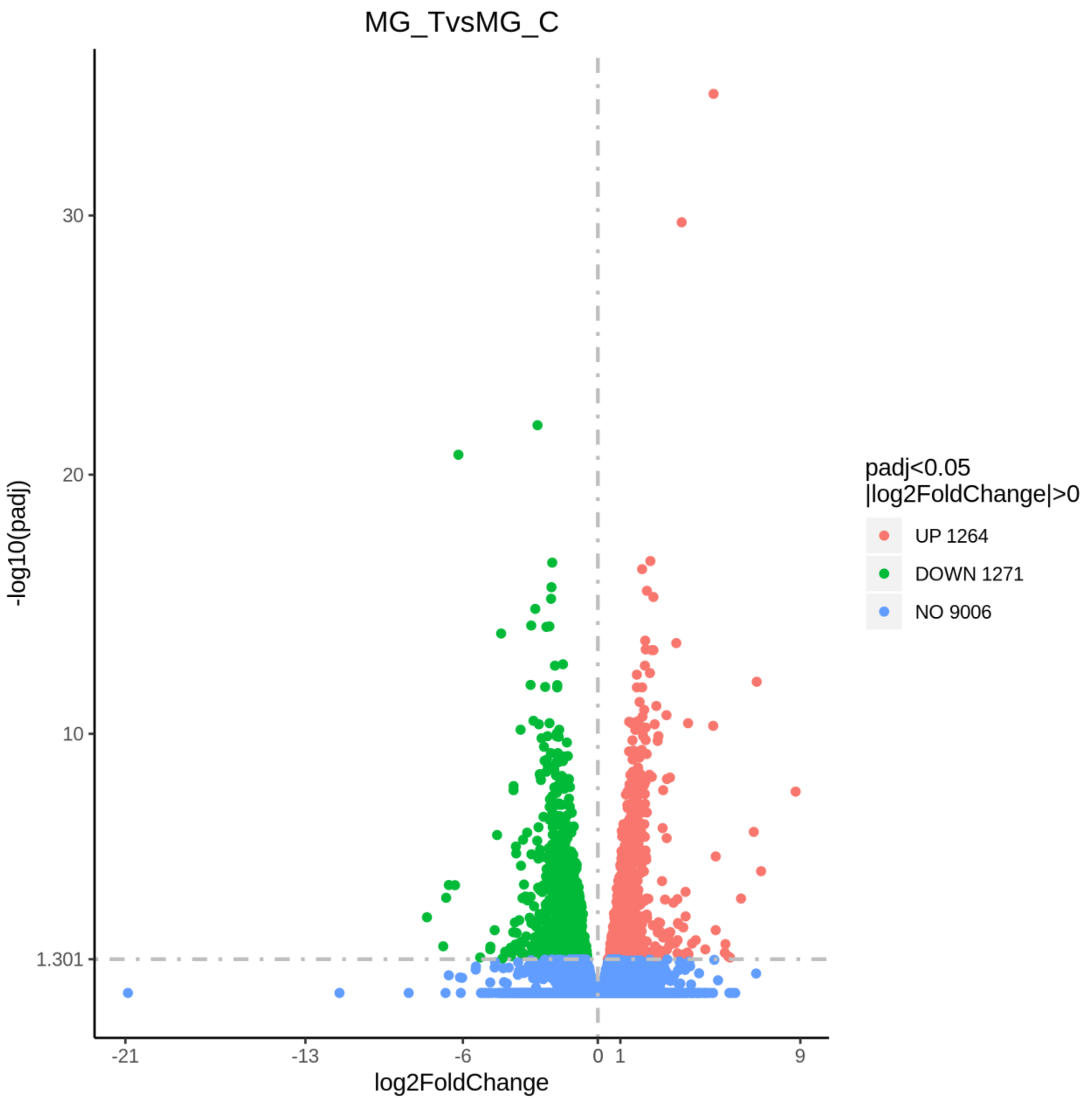

Figure S1. Volcano map of the DEGs

Note: The abscissa represents the fold change of gene expression in different experimental groups, and the ordinate represents the statistical significance of gene expression changes. Each point in the figure represents each gene, and the red and green points represent genes that are significantly up-regulated and down-regulated.

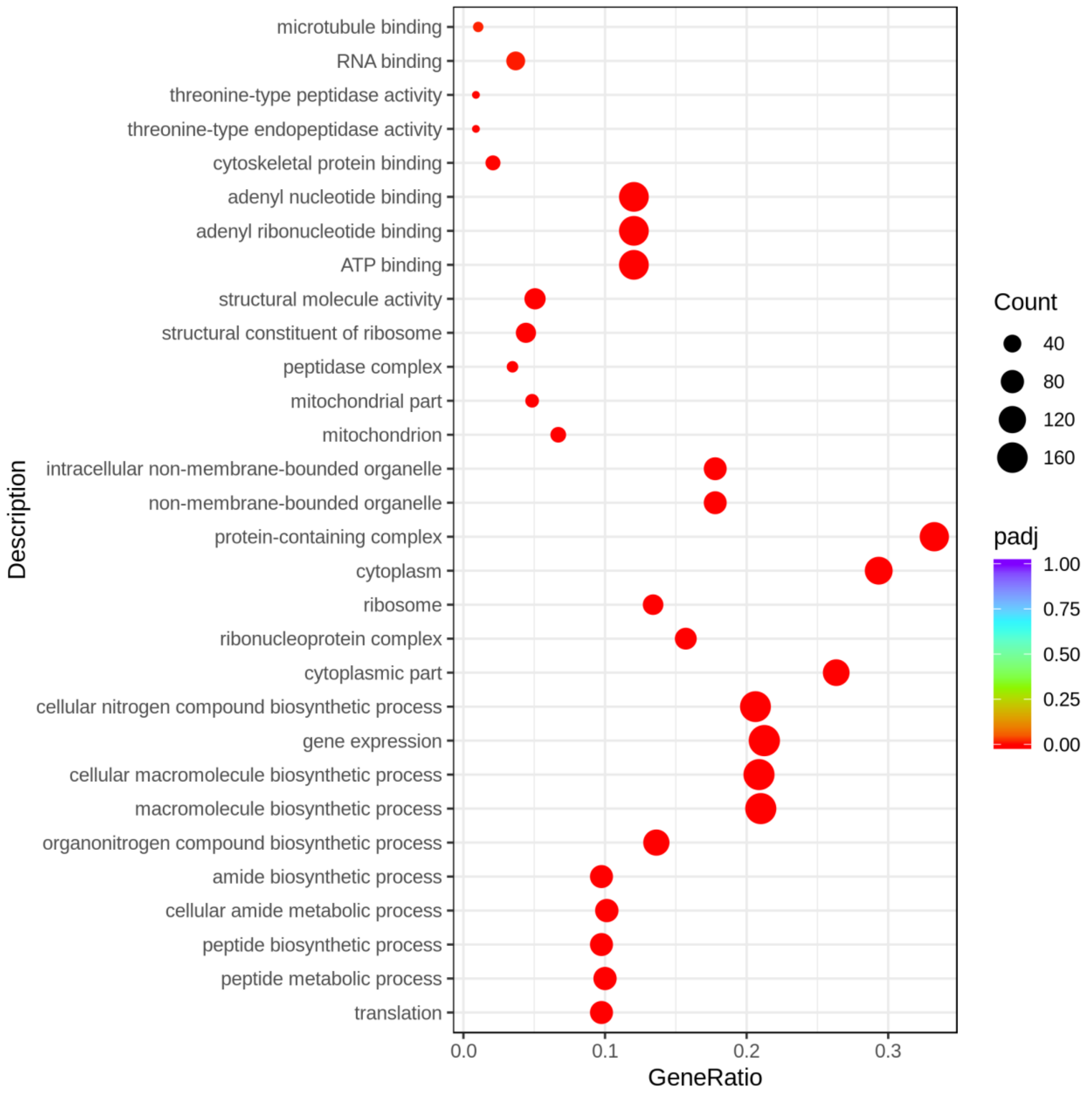

Figure S2. Scatter plot of GO enrichment analysis

Note: The abscissa in the figure is the ratio of the number of differential genes annotated to the GO term to the total number of differential genes, the ordinate is the GO term, the size of the scatter points represents the number of genes annotated to the GO term, and the color from red to purple represents rich the saliency size of the set.

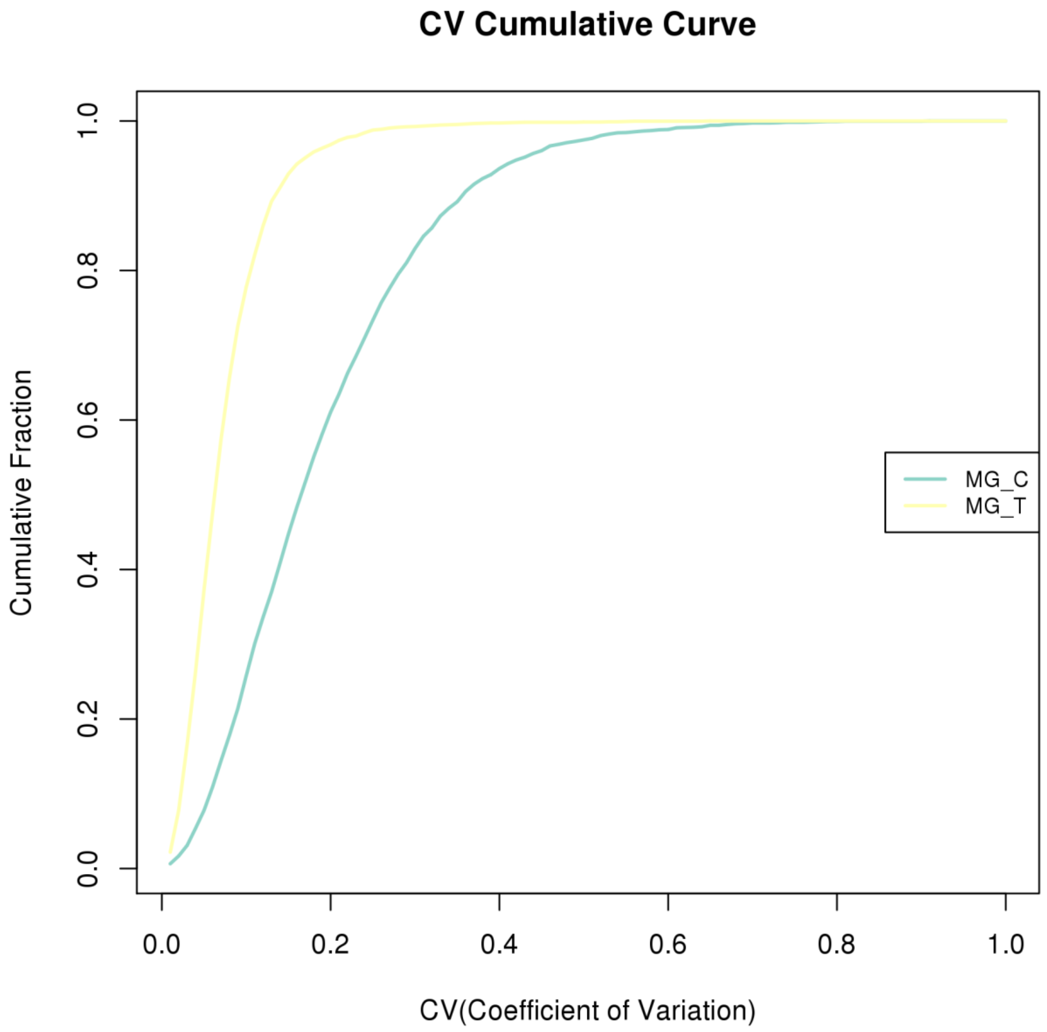

Figure S3. Repeatable CV analysis

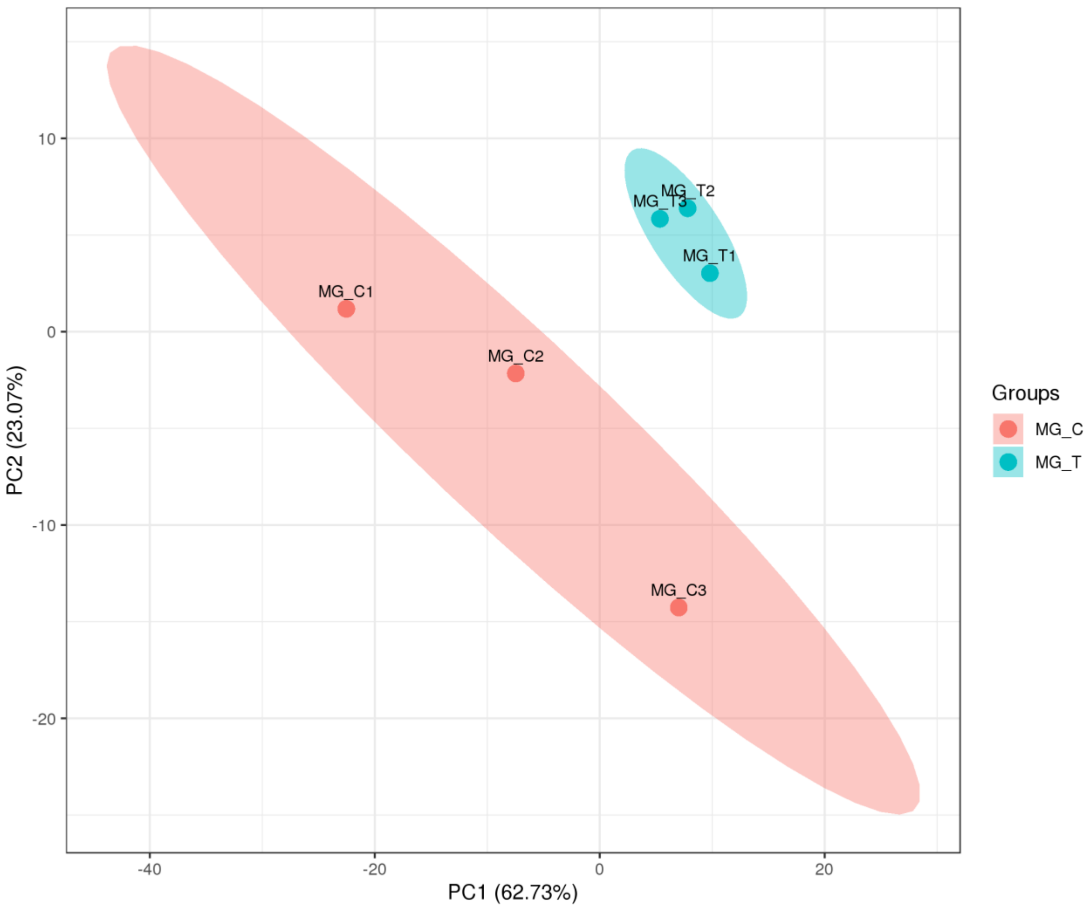

Figure S4. PCA analysis

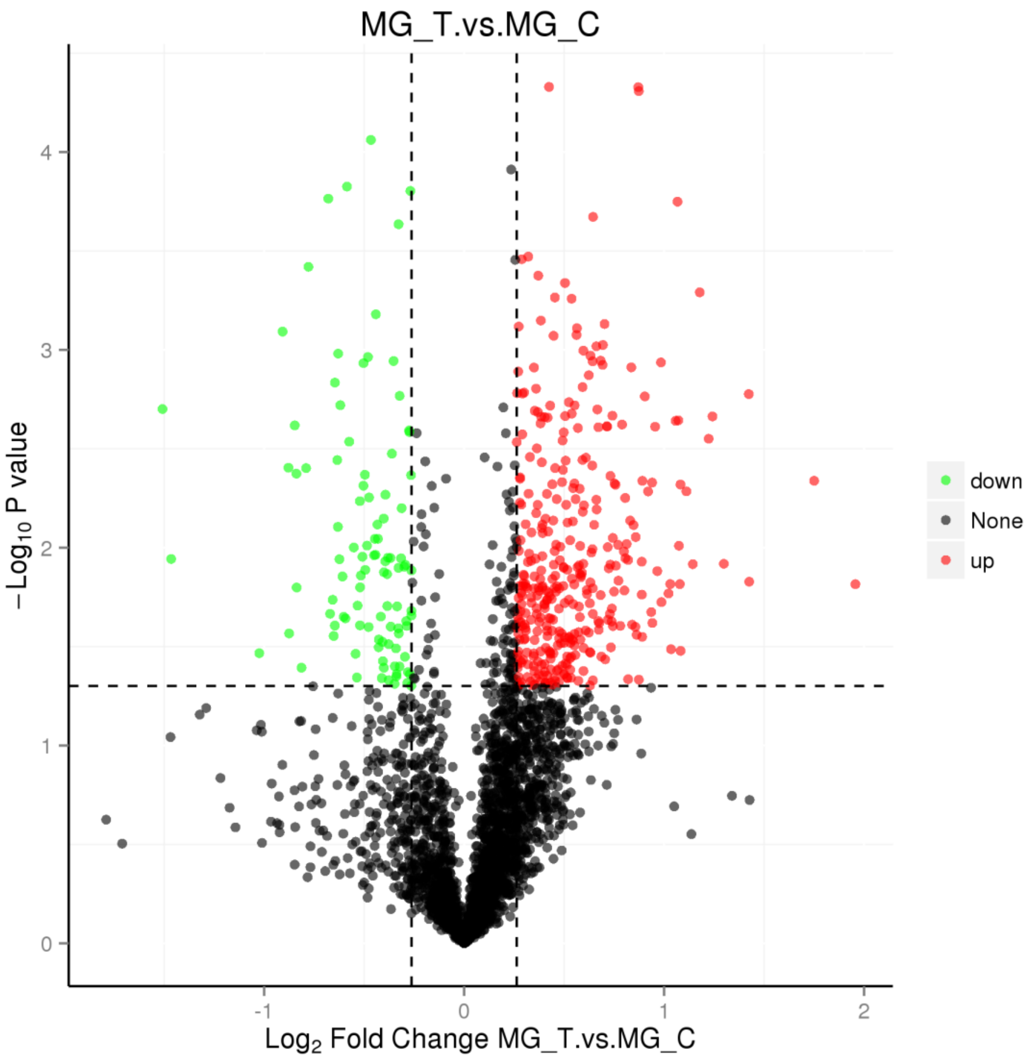

Figure S5. Volcano map of the DEPs

Note: The abscissa represents the fold change of protein expression in different experimental groups, and the ordinate represents the statistical significance of protein expression changes. Each point in the figure represents each protein, and the red and green points represent significantly up-regulated and down-regulated proteins.

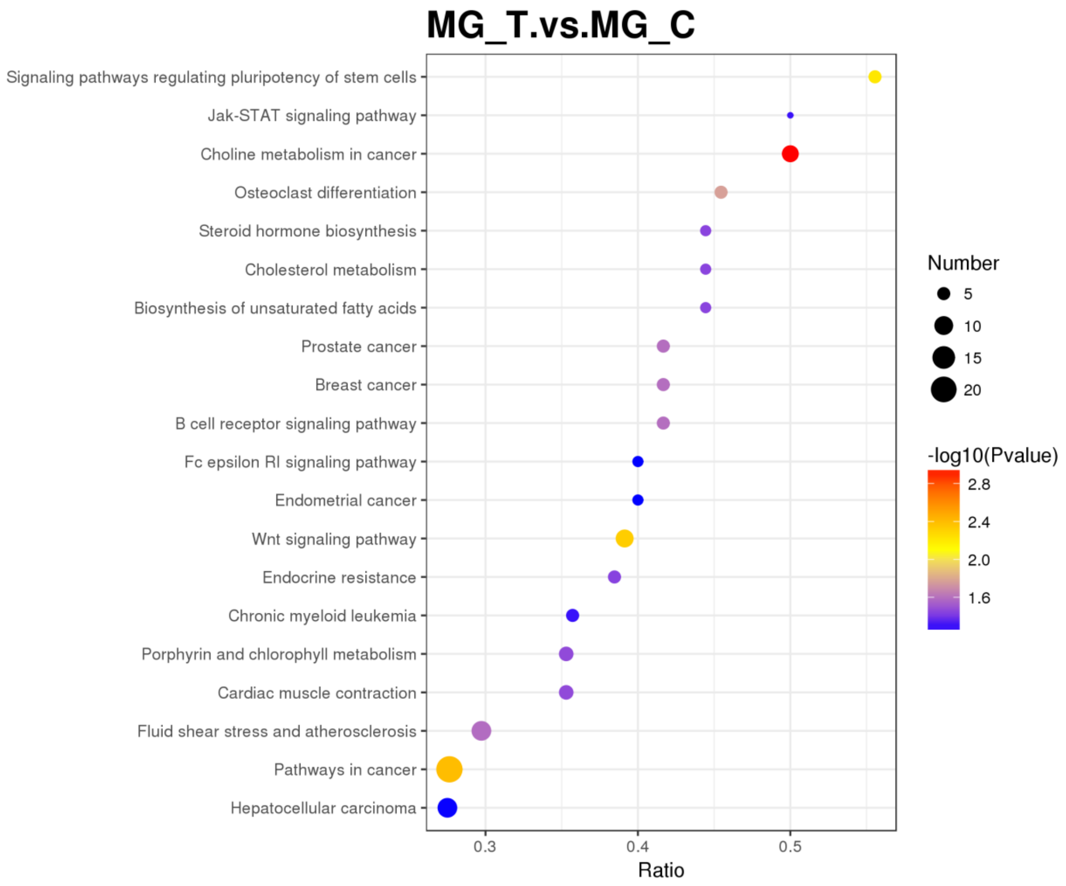

Figure S 6. Scatter plot of DEPs' KEGG pathway analysis

Note: The abscissa in the figure is the ratio of the number of differential proteins in the corresponding pathway to the total number of proteins identified in the pathway. The larger the value, the enrichment of differential proteins in the pathway is higher. The color of the point represents the P-value of the hypergeometric test. The size of the dot represents the number of differential proteins in the corresponding pathway.

Table S1 10 Primer sequences of DEGs for qRT-PCR verification

Genes name		Primer sequence (5'-3')
evm.TU.Hic_asm_6.736	F	ACCGAGCGAATATAATGTTG
	R	TGTCTGAGCCATCTACGA
evm.TU.Hic_asm_7.386	F	GATGCCGTCAAGGTGTATA
	R	TAGTGGTCGCTGCTACAT
evm.TU.Hic_asm_9.807	F	AGACGGTGATACGGTGAA
	R	CCATCGGCATCCTTACTG
evm.TU.Hic_asm_5.865	F	GAGAGGCTACCGTTCAAG
	R	TTCCATACCAGCGATGATC
evm.TU.Hic_asm_9.444	F	TCTCGCTTCACATCTCATC
	R	GCCTGACTAGAACGCATT
evm.TU.Hic_asm_4.422	F	CGTATCGCTCCTCAATGTT
	R	CCTGTAATCCTTCGTGGTAA
evm.TU.Hic_asm_6.129	F	ACACCAGCACAGTTACATAA
	R	CGCCTTCCATTAGACTCTC
evm.TU.Hic_asm_9.336	F	GCGTCAGCAATCATTCCT
	R	CCGAGAAGCAGCAGAATT
evm.TU.Hic_asm_11.708	F	GCTGAACGCTGAAGAGTTCG
	R	GAACGAGACCGCTTGATTTC
evm.TU.Hic_asm_2.621	F	TCTCGTATTAACGCACTGA
	R	GACTCATCCTCCATTCCTTA

Table S2 7 Primer sequences of DEGs for qRT-PCR verification

Genes name		Primer sequence (5'-3')
evm.TU.Hic_asm_3.922	F	GCTGGGAACTGAGGAATTGT
	R	TAACGCAGAAGACGGTCAAG
evm.TU.Hic_asm_3.207	F	GATGCCGACATTGAACTTGA
	R	TGCTTCCGATATGTTGTATGG
evm.TU.Hic_asm_3.1077	F	CGGAACAAGATGGCACTTC
	R	CATTGCTTCGTAGGATTCGTA
evm.TU.Hic_asm_13.549	F	CGCACGACGATACGATACTC
	R	AGCGGTAGAGTCTCATTGTG
evm.TU.Hic_asm_11.737	F	CACTGGTCTGGTTGGTACTC
	R	TTGGCTCGGATCTCAATACG
evm.TU.Hic_asm_5.10	F	ACGCTATGCCAGATTACACT
	R	CACGACTCCACATAACTCAC
evm.TU.Hic_asm_5.109	F	GGCAGGTTAGTCTATGTGAA
	R	TGGTCCATTCTAGGAAGTCT

Table S3. Transcriptome and proteome GO enrichment DEGs/DEPs statistics table

		Prot-go	Tran-go	Total
DEGs/DEPs		434	2535	2969
Annotated Genes/ protein		290	581	871
GO term	Biological Process	151	405	556
	Cellular Component	106	196	302
	Molecular Function	219	313	532
	Total	476	914	1390
